# Accessory Chromosome-Acquired Secondary Metabolism in Plant Pathogenic Fungi: The Evolution of Biotrophs Into Host-Specific Pathogens

**DOI:** 10.3389/fmicb.2021.664276

**Published:** 2021-04-23

**Authors:** Thomas E. Witte, Nicolas Villeneuve, Christopher N. Boddy, David P. Overy

**Affiliations:** ^1^Agriculture and Agri-Food Canada, Ottawa Research and Development Centre, Ottawa, ON, Canada; ^2^Department of Chemistry and Biomolecular Sciences, University of Ottawa, Ottawa, ON, Canada

**Keywords:** accessory chromosomes, secondary metabolites, biosynthetic gene clusters, *Alternaria* host specific toxins, metabolomics, RIP, *Alternaria* pathotypes, *Fusarium*

## Abstract

Accessory chromosomes are strain- or pathotype-specific chromosomes that exist in addition to the core chromosomes of a species and are generally not considered essential to the survival of the organism. Among pathogenic fungal species, accessory chromosomes harbor pathogenicity or virulence factor genes, several of which are known to encode for secondary metabolites that are involved in plant tissue invasion. Accessory chromosomes are of particular interest due to their capacity for horizontal transfer between strains and their dynamic “crosstalk” with core chromosomes. This review focuses exclusively on secondary metabolism (including mycotoxin biosynthesis) associated with accessory chromosomes in filamentous fungi and the role accessory chromosomes play in the evolution of secondary metabolite gene clusters. Untargeted metabolomics profiling in conjunction with genome sequencing provides an effective means of linking secondary metabolite products with their respective biosynthetic gene clusters that reside on accessory chromosomes. While the majority of literature describing accessory chromosome-associated toxin biosynthesis comes from studies of *Alternaria* pathotypes, the recent discovery of accessory chromosome-associated biosynthetic genes in *Fusarium* species offer fresh insights into the evolution of biosynthetic enzymes such as non-ribosomal peptide synthetases (NRPSs), polyketide synthases (PKSs) and regulatory mechanisms governing their expression.

## Introduction

Some ascomycete lineages are prolific producers of secondary metabolites (natural products), many of which have associated activity in biological systems. Large scale screening and characterization of fungal extracts has been carried out for over half a century by various industries (including pharmaceutical) and as a result, a considerable breadth of the chemical space associated with *in vitro* culturing of filamentous ascomycetes has been probed for associated biological activity (Bills et al., 2009). However, with the advent of modern genome sequencing technologies, it has become evident that many secondary metabolite biosynthetic gene clusters are associated with unknown products and are not expressed under axenic conditions (Mao et al., 2018). The fungal natural product community is therefore focused on unlocking and understanding this “cryptic” metabolome and strive to link secondary metabolite products with their respective biosynthetic gene clusters.

In agriculture, fungal secondary metabolites produced by plant pathogens and spoilage fungi can be mycotoxins acting as virulence factors that directly impact crop yields and market suitability. Fungal plant pathogens with biotrophic/necrotrophic and saprophytic lifestyles (such as species from the genera of *Fusarium* and *Alternaria*), produce a broad diversity of secondary metabolites. However, secondary metabolism can vary within species populations of some plant pathogens. One reason for observed intra-species variations in secondary metabolism arises from the presence of lineage-specific accessory chromosomes (ACs) within the genome of select strains that encode secondary metabolite biosynthetic gene clusters.

This review focuses exclusively on secondary metabolism (including mycotoxin biosynthesis) associated with ACs in filamentous fungi. We fill in historical gaps and provide a current overview of both the pathways and associated gene clusters involved in the biosynthesis of host specific toxins and other secondary metabolite virulence factors associated with ACs to date. Examples of AC-associated toxin biosynthesis are numerous among *Alternaria formae speciales* (*Alternaria* f. sp.) and recent genomic studies of *Fusarium* species have also detected biosynthetic enzymes on accessory chromosomes. Enzymes of interest include non-ribosomal peptide synthetases (NRPSs), polyketide synthases (PKSs), terpene synthases and the regulatory mechanisms governing their expression. Relationships between ACs and secondary metabolite biosynthesis are discussed, including evidence for horizontal chromosome transfer of ACs between fungi, the relevance of ACs providing a niche for rapid multiplication and diversification of secondary metabolite biosynthesis genes, the implications of regulatory “cross talk” between core chromosomes and ACs, and the role of “repeat-induced point mutation” in detecting and eliminating duplicated genes in the context of AC-associated gene duplications. The utility of long-read sequencing technology to elucidate the structure of genomes through “closed” or “telomere to telomere” chromosomal assemblies is highlighted as essential for the study of ACs, greatly improving analyses of secondary metabolite gene clusters. Finally, a “top down—bottom up” screening strategy coupling genomics based approaches with untargeted metabolomics is presented, where the production of unique secondary metabolite products within species populations can be linked with biosynthetic gene clusters associated with ACs.

## What Are Fungal Accessory Chromosomes?

The term “accessory chromosome” (or AC) has been adopted by researchers working on a variety of different Ascomycete genera and conceptually encompasses the terms “type B,” “supernumerary,” or “lineage-specific” chromosomes used in the study of plants, animals and fungal species (Covert, 1998; Croll and McDonald, 2012). We have decided to use the term AC in this review as it is consistent with previous fungal research and consensus on the different terms’ proper usage is lacking in the literature. Fungal ACs are generally small, lineage- or pathotype-specific chromosomes that exist in addition to the “core” chromosomes of a species and are not considered essential to the survival of the organism. Among some pathogenic fungi, the concept of an AC is nuanced by the presence of virulence factor genes involved in pathogenicity and/or secondary metabolite production, which play an important role in niche plant invasion and adaptation (Croll and McDonald, 2012). ACs conferring adaptive advantages such as pathogenicity are sometimes described as “conditionally dispensable” chromosomes (popularized by studies of *Alternaria* species; Tsuge, 2019) or “pathogenicity” chromosomes (Ma et al., 2010; van Dam et al., 2017; Li et al., 2020). ACs are of particular interest in fungal pathogen research due to their capacity for horizontal transfer between strains, which has been experimentally demonstrated to increase pathogen competence in previously deficient strains (He et al., 1998; Akagi et al., 2009; Ma et al., 2010; Shahi et al., 2016; van Dam et al., 2017; Li et al., 2020). Pathogenicity traits associated with ACs have given rise to *formae speciales* or pathotypes, causal agents of host specific plant diseases.

Fungal ACs display key structural and genetic differences compared to core chromosomes. ACs are distinguished in some species by their low gene density, high level of transposable element (TE) distribution, epigenetic markers, and non-Mendelian inheritance patterns [observed either by spontaneous loss of ACs or more recently by meiotic drive within populations; (Habig et al., 2018)]. ACs vary in size and distribution among individuals of the same species and many do not appear to be under strong selective pressure, exhibiting high levels of sequence polymorphisms or disruption of chromosomal collinearity (for recent reviews of fungal ACs we direct the reader to Bertazzoni et al., 2018 and Yang et al., 2020). Nevertheless, the association of certain ACs with pathogenicity indicates ACs are not trivial to the evolution and survival of fungi outside of the laboratory. ACs contribute to the compartmentalization of genomic regions associated with high repeat content and accelerated evolution of virulence factors as proposed in the “two-speed genome” concept of fungal pathogen adaptation (Raffaele et al., 2010; Croll and McDonald, 2012).

The presence of virulence factors on fungal ACs, and their distribution within populations, is highly relevant to the study of fungal plant pathogens. The best-characterized virulence factors associated with plant pathogen ACs fall into at least three groups: small secreted effector proteins such as the secreted in xylem (SIX) genes in *Fusarium oxysporum* (Rep et al., 2004; Houterman et al., 2007; Schmidt et al., 2013), detoxification genes such as the pisatin demethylase gene *pda6* in the pea-pathogenic strain *Fusarium solani* f. sp. *pisi* (Han et al., 2001), and toxin biosynthesis genes such as AM-toxin genes conferring pathogenicity of *Alternaria alternata* f. sp. *mali* on apple trees (*Malus domestica*) (Johnson et al., 2000; Andersen et al., 2006; Harimoto et al., 2007). As more ACs are identified and analyzed, the list of associated genes contributing to pathogenicity expands, as is our understanding of the interplay between core and accessory chromosomes and the role ACs play in coordinating regulatory shifts in their expression.

## Secondary Metabolism Linked With Accessory Chromosomes

**FIGURE 1:**
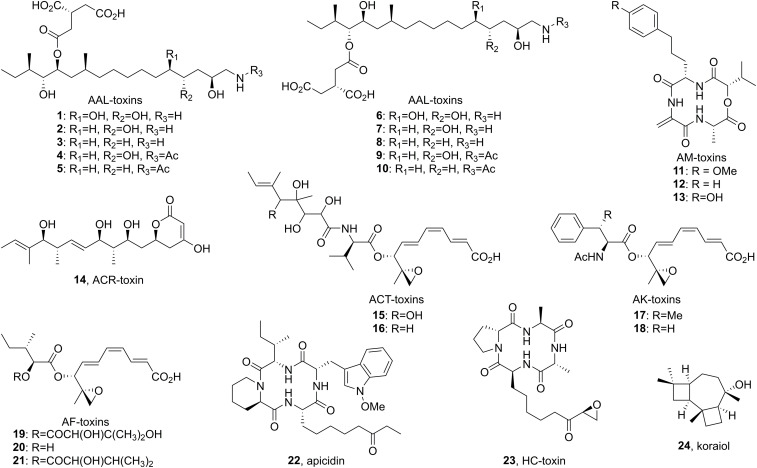
Structure of host specific toxins (1–21) and other secondary metabolites associated with accessory chromosomes (22–24).

In some instances, only a small subsection of a species population can infect a specific host due to the expression of secondary metabolite biosynthetic gene clusters residing on ACs. These secondary metabolites are referred to as host specific toxins (Figure 1). Historically, much research interest has been invested into understanding the production of host specific toxins, including structure determination, genes associated with their biosynthesis and the association of biosynthetic genes with ACs. For example, several unique populations of *Alternaria alternata* cause disease on niche plant hosts due the production of host specific toxins (described below). The taxonomy of small spored *Alternaria* species is notoriously convoluted, in part due to strains that are morphologically indistinguishable but produce different host specific toxins and thus have a unique host range. Differing usages of either the term pathotype or *formae speciales* (or combinations of the two) within the genus *Alternaria* exist (Akimitsu et al., 2014; Woudenberg et al., 2015; Armitage et al., 2020). The majority of research on host specific toxins in *Alternaria* was carried out prior to the advent of next generation and long-read sequencing tools. As older studies lack high quality telomere-telomere genomes, localization of host specific toxin biosynthetic gene clusters to ACs was typically based by electrophoretic karyotyping and visualized using a Southern blot probe. To fill in existing gaps in the literature, we illustrate *Alternaria* biosynthetic pathways and summarize the evidence confirming the localization of their associated biosynthetic genes to ACs.

### AAL-Toxin

**FIGURE 2:**
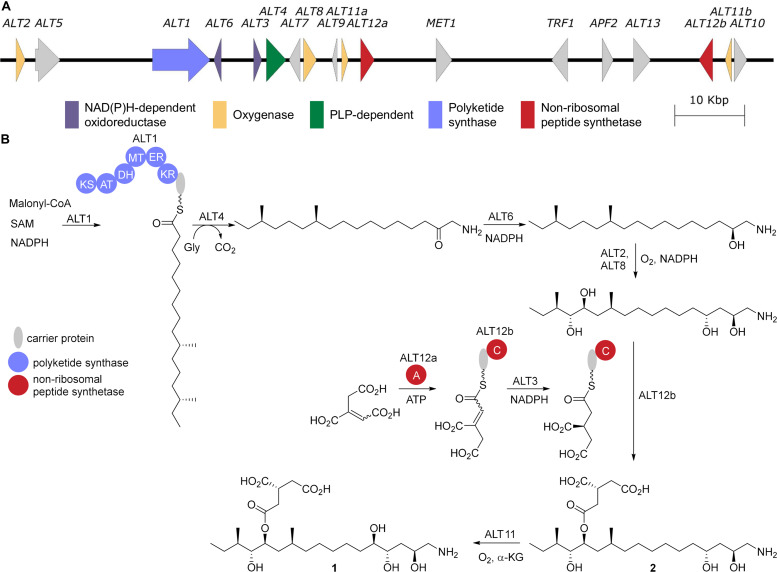
AAL-toxin biosynthesis. **(A)** The gene cluster for AAL-toxin biosynthesis from *A. alternata* (AB969680). **(B)** The proposed biosynthesis for AAL-toxin TA1 based on homology to fumonisin biosynthesis. KS, β-ketosynthase; AT-acyltransferase; KR, β-ketoreductase; DH, dehydratase; ER, enoylreductase; MT methyltransferase; A, adenylation; C, condensation.

The tomato pathotype of *A. alternata* (*Alternaria alternata f.* sp. *lycopersici*), which causes Alternaria stem canker on tomato, produces 5 regioisomeric pairs of AAL-toxins: TA, TB, TC, TD, and TE (Caldas et al., 1998; Figure 1). While direct characterization of AAL-toxin biosynthesis is limited, the well-characterized and highly related fumonisin biosynthetic pathway serves as a reliable guide for AAL-toxin biosynthesis (Figure 2). Homology between the two biosynthetic gene clusters has been confirmed via a complementation experiment where the highly reducing PKS responsible for formation of the polyketide core of AAL-toxin, *ALT1*, was shown to complement *FUM1*, the PKS gene linked to the biosynthesis of fumonisin, a sphingolipid inhibitor produced by *Fusarium verticillioides* (Zhu et al., 2008). Thus, based on the homology to fumonisin biosynthesis, ALT1 can be inferred to generate a 16 carbon long saturated enzyme-linked thioester that is off-loaded from the PKS by the 2-oxoamino synthase ALT4 via a PLP-dependent decarboxylative condensation with glycine (Du et al., 2008). The product is then likely reduced by the NADPH-dependent short chain dehydrogenase ALT6 (Butchko et al., 2003; Yi et al., 2005), in an overall process akin to dihydrosphingosine biosynthesis (Gault et al., 2010). Oxidation at C13 and C14 by the cytochrome p450 enzyme ALT2 and C4 by ALT8 generate an oxidized intermediate, lacking the tricarballyl group and C5 oxidation of the final product (Bojja et al., 2004). The tricarballyl group is generated from aconitate, likely transported from the mitochondria by ALT9, which is converted into the AMP ester via ALT12, and loaded onto the carrier protein of ALT12b. Reduction of the enoyl group by the dehydrogenase ALT3 generates the (*R*)-configured tricarballyl-enzyme intermediate that is then transferred, via the condensation domain of ALT12b, onto the polyketide chain at either the C14 or C15 alcohol (Lia et al., 2013). The final biosynthetic step is the dioxygenase-catalyzed C5 oxidation by ALT11a (Ding et al., 2004). Among the AAL toxins, TA, TB, TC, and TE all differ in the extent of the oxidation of the polyketide core and the presence or absence of an N-acetate and each of the AAL toxins exist as regioisomeric pairs that differ in the location of the tricarballylate ester, which is on either the C13 (e.g., TA1) or C14 (e.g., TA2) alcohol (Figure 1).

Using electrophoretic karyotyping and Southern blot probe labeling experiments, the PKS gene *ALT1* was found to be unique to isolates of the tomato pathotype of *A. alternata* and absent in non-pathogenic strains—*ALT1* solely resides on a 1.0 Mb AC in producing strains (Akagi et al., 2009). When produced within the appropriate host plant cell, the AAL-toxins affect the endoplasmic reticulum and mitochondria by preventing the enzyme sphingosine-N-acyltransferase (ceramide synthase) from contributing to the production of ceramide-containing lipids (Gilchrist, 1998). This initiates premature programmed cell death via the accumulation of nitric oxide, reactive oxygen species, and other signaling molecules (Wolpert et al., 2002; Gechev et al., 2004) of the five AAL-toxins isoforms, TA-toxin is the most toxic to tomato and is produced in the highest quantity by producing strains (Spassieva et al., 2002). Interestingly, while the ALT biosynthetic gene cluster encodes an additional copy of ceramide synthase, ALT7, this is not part of a self-resistance mechanism as an *ALT7* deletion has no effect on growth rate of the pathogenic strain (Kheder and Akagi, 2012).

### ACR-Toxin

The rough lemon pathotype of *A. alternata* (*A. alternata f.* sp. *citri*), is the causal agent of Alternaria leaf spot of rough lemon (*Citrus jambhiri* Lush.) and rangpur lime (*C. limonia* Osbeck), and causes the formation of lesions on, and the abscission of, leaves and immature fruit (Peever et al., 2004). The known host range of the *A. alternata* rough lemon pathotype is rather narrow and attributed to the production of a host specific toxin, ACR-toxin (Masunaka et al., 2005). Within the cell, ACR-toxin affects the mitochondria, which it renders dysfunctional by the uncoupling of oxidative phosphorylation, leakage of NAD + cofactor from the TCA cycle, and a loss of membrane potential (Kohmoto et al., 1984; Akimitsu et al., 1989; Meena et al., 2017).

**FIGURE 3:**
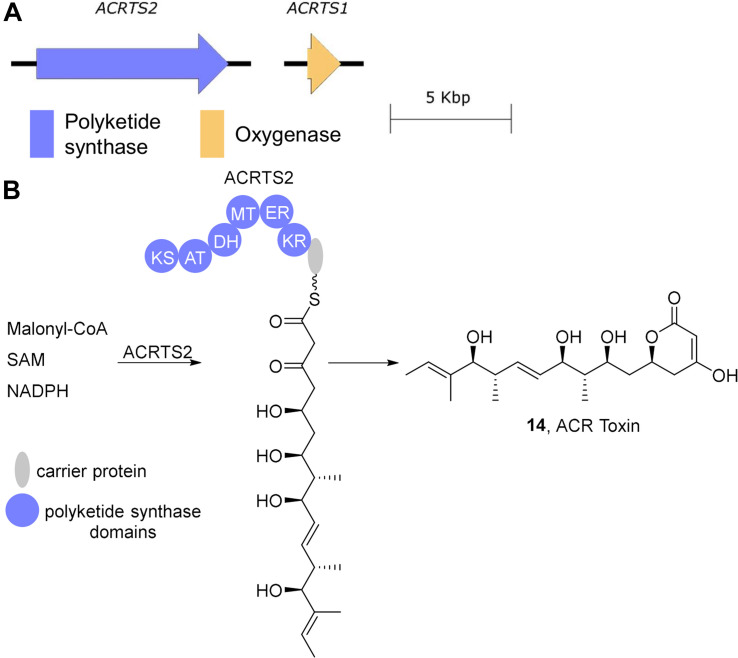
ACR-toxin biosynthesis. **(A)** The ACRTS1 and ACRTS2 genes involved in ACR-toxin biosynthesis. **(B)** ACR-toxin is generated from ACRTS2. KS, β-ketosynthase; AT, acyltransferase; KR, β-ketoreductase; DH, dehydratase; ER, enoylreductase; MT, methyltransferase.

ACR-toxin is a polyketide consisting of an α-dihydropyrone ring in a 19-carbon polyol (Gardner et al., 1985; Figure 3). The highly reducing type I polyketide synthase encoded by *ACRTS2* is likely responsible for its biosynthesis as both RNA silencing of ACRTS2 and homologous recombination-based gene disruption abolishes ACR toxin production (Izumi et al., 2012a, b). A second gene *ACRTS1* has also been proposed to be required for ACR-toxin production since disruption of ACRTS1 leads to a decrease in toxin production (Izumi et al., 2012a). While this gene was initially proposed to play a role in generating the dihydropyrone (Izumi et al., 2012a), sequence analysis shows that it encodes a cytochrome p450, which is inconsistent with this proposed function. It thus remains to be seen what, if any, direct role ACRTS1 plays in ACR-toxin biosynthesis. ACRTS2 was confirmed to reside on a 1.5 Mb AC of rough lemon pathotype strains by electrophoretic karyotyping and Southern blot probe labeling experiments and ACRTS2 was shown to be absent in non-producing stains of *A. alternata* (Masunaka et al., 2005; Izumi et al., 2012b).

### ACT-, AF-, and AK-Toxin

ACT-, AF-, and AK-toxins are related toxins with a common core polyketide. ACT-toxin is produced by the tangerine pathotype of *A. alternata* (*A. alternata f.* sp. *citri*) that affects grapefruit, hybrids of grapefruit and tangerine, tangerine and sweet orange (Timmer et al., 2003; Ma et al., 2019). Japanese pear black spot disease is caused by *A. gaisen* (syn. *A. alternata* Japanese pear pathotype) due to the production of the host specific AK-toxin (Tsuge et al., 2013). The strawberry pathotype of *A. alternata* (*A. alternata f.* sp. *fragariae*) affects both strawberry and pear due to the production of several host specific toxins (AF-toxins I, II, and III) (Tsuge et al., 2013). ACT-, AF-, and AK-toxins all act to depolarize the cellular plasma membrane resulting in invagination, fragmentation and vesiculation, causing cell necrosis in susceptible leaves (Kohmoto, 1993; Park and Ikeda, 2008).

These toxins all share a 9,10-epoxy-8-hydroxy-9-methyl-decatrienoic acid (EDA) moiety (Kohmoto et al., 1985; Nakatsuka et al., 1986; Kohmoto, 1993; Akimitsu et al., 2003) and differ based on the acyl groups attached to the C8 hydroxyl of the EDA moiety. Biosynthetic gene clusters encoding a PKS have been identified for ACT-, AF-, and AK-toxin biosynthesis and archived in MIBiG, the repository for biosynthetic gene clusters of known function (Tanaka and Tsuge, 2000; Ruswandi et al., 2005; Miyamoto et al., 2010; Takaoka et al., 2014; Kautsar et al., 2020). However, only the PKS from AF- and AK-toxin biosynthesis show significant homology. With a common polyketide core, all three gene clusters would be expected to have at least one homologous PKS. Further examination of the PKS from AF- and AK-toxin biosynthesis shows that the AK-toxin PKS (AFT9h) is pseudogenized. Comparison of the catalytic domains from the AF- and AK-toxin PKSs (AFT9-1 and AFT9h, respectively) show typical highly reducing PKS modules with a C-terminal carnitine O-palmitoyltransferase. Presumably this acyltransferase domain is responsible for off-loading of the completed polyketide, akin to side chain installation in lovastatin biosynthesis (Xie et al., 2009). However, transacylation would produce an ester, inconsistent with the structures of AF-, and AK-toxins that terminate in a carboxylic acid. Potentially this PKS could play a role in side chain installation in AF-toxin biosynthesis, via transacylation of the PKS bound side chain onto the EDA core. In AK-toxin biosynthesis, an aminoacyl chain is added to the C8 hydroxyl group of EDA, thus no transacylating PKS is required, consistent with the pseudogenization of this PKS in the MIBiG AK-toxin biosynthetic gene cluster.

A unique aspect of the EDA core polyketide is its epoxidized isopropyl tail. This unit could arise from either a 2-hydroxyisovalerate or an α,β-unsaturated isovalerate starter unit (Ray and Moore, 2016) for the PKS. Disruption of *AFTS1*, which encodes a 2-ketoisovalerate reductase and thus should eliminate 2-hydroxyisovalerate formation, did not abolish AF-toxin II biosynthesis (Ito et al., 2004). This strongly suggests that 2-hydroxyisovalerate is not the required starter unit for the core polyketide production.

**FIGURE 4:**
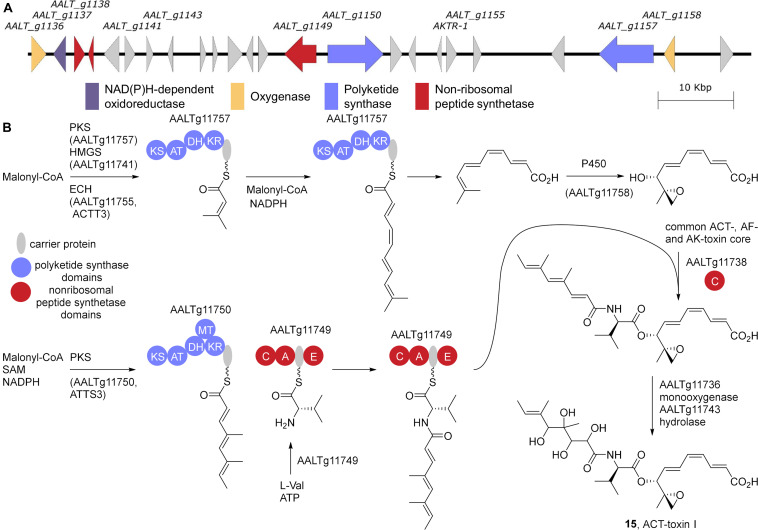
ACT-toxin biosynthesis. **(A)** The gene cluster from *A. alternata* Z7 (LPVP01000048). **(B)** The proposed biosynthesis of the ACT-, AF-, and AK-toxin core and the addition of the ACT-toxin I side chain. KS, β-ketosynthase; AT, acyltransferase; KR, β-ketoreductase; DH, dehydratase; MT, methyltransferase; A, adenylation; C, condensation; E, epimerization.

Intriguingly, a larger gene cluster spanning 90 kb of the ACT-toxin producing tangerine pathotype *A. alternata* Z7 genome was proposed to encode the ACT-gene cluster (Wang et al., 2016; Figure 4). Of particular note in this cluster is the presence of a HMG-CoA synthase and an enoyl-CoA hydratase, that are suggestive of a potential mechanism for installing an α,β-unsaturated isovalerate via a β-branching type mechanism (Walker et al., 2020). Results from isotope labeling experiments with ^13^C labeled acetate are consistent with this hypothesis (Nakatsuka et al., 1990). Additionally, disruption of the two copies of enoyl-CoA hydratase encoding *ACTT6* in *A. alternata* SH20 eliminates ACT-toxin production (Miyamoto et al., 2009). Lastly, while an HMG-CoA synthase has not yet been found in the AF-toxin biosynthetic gene cluster, one is associated with AK-toxin biosynthesis, AKT4 (Takaoka et al., 2014). Based on these data, the PKS AALTg11757 in combination with the HMG-CoA synthase AALTg11755 (previously identified as ACTT3; Miyamoto et al., 2008) and the cytochrome p450 AALTg11750 are responsible for assembling the common core of ACT-, AF-, and AK-toxin (Figure 4). Presumably, homologs of all these genes will be identified in AF- and AK-toxin producers as well. PKS AALTg11750 (ACTTS3) (Miyamoto et al., 2010) in combination with the NRPS AALTg11749 generate the side chain, which is added to the core via the condensation domain AALTg11738. Lastly epoxidation of the side chain by the Flavin monooxygenase AALTg11736 followed by epoxide hydrolysis via AALTg11743 (ACTT2) (Miyamoto et al., 2008) gives ACT-toxin I, **15**, as shown.

While the proposed biosyntheses of these toxins are yet to be fully characterized, strong evidence shows that their biosynthetic gene clusters are all present on ACs. For example, ACT-toxin associated genes *ACTT1* and *ACTT2* were shown by pulsed-field gel electrophoresis and Southern hybridization to be present only on the 1.9 Mb chromosome in the tangerine pathotype *A. alternata* SH20 (Masunaka et al., 2005). Similarly, a 32kb cosmid clone from the 1.05 Mb AC of the strawberry pathotype *A. alternata* NAF8 encoded the AF-toxin associated genes *AFT1*, *AFTR* and *AFT3* (Hatta et al., 2002). Lastly probes for AK-toxin genes *AKT1, AKT2, AKTR*, and *AKT3* were shown to hybridize to the 4.1 Mb AC of the pear pathotype *A. alternata* A15 (Tanaka and Tsuge, 2000).

### AM-Toxin

AM-toxin is a four-residue cyclic depsipeptide produced by a pathotype of *A. alternata* (*Alternata f.* sp. *mali*) causing Alternaria blotch on a narrow range of apple cultivars world-wide (Ueno et al., 1975; Kohmoto et al., 1977; Filajdic and Sutton, 1991). AM-toxin affects both the chloroplast and plasma membrane of leaf cells, where the toxin inhibits photosynthetic CO_2_ fixation and induces electrolyte loss, resulting in tissue damage of susceptible leaves (Kohmoto et al., 1983; Shimomura et al., 1991; Zhang et al., 2015).

**FIGURE 5:**
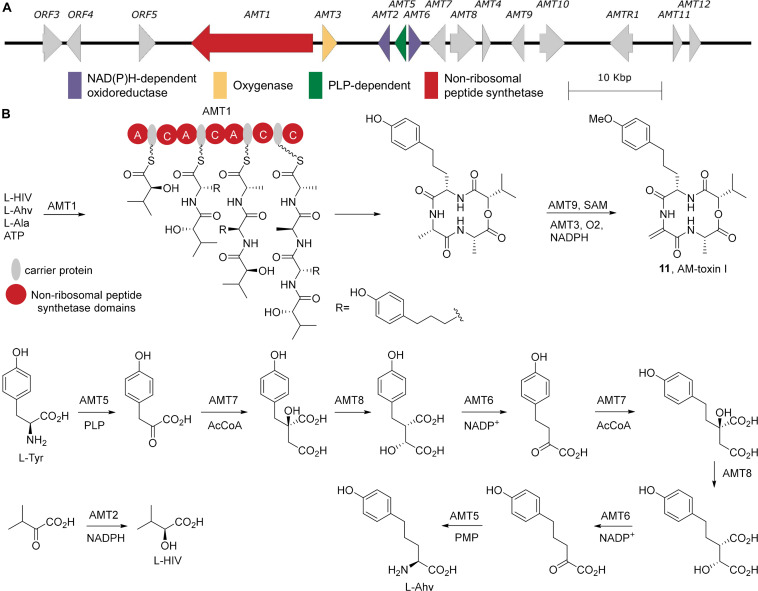
AM-toxin Biosynthesis. **(A)** The biosynthetic gene cluster from *A. alternata* strain NBRC 8984 (AB525198) **(B)** The biosynthetic pathway for AM-toxin I and its non-proteinogenic building blocks. A, adenylation; C, condensation.

AM-toxin is a non-ribosomal peptide and the NRPS associated with its biosynthesis, AMT1, has been identified (Johnson et al., 2000). In addition, a 2-ketoisovalerate reductase (AMT2), a cytochrome p450 (AMT3), and a thioesterase (AMT4) were associated with AM-toxin biosynthesis (Ito et al., 2004; Harimoto et al., 2007). Sequencing of cDNA clones that hybridized with the chromosome containing AMT1 and AMT2 identified a number of key metabolic genes required for the complete biosynthesis of AM-toxin (Harimoto et al., 2007), all of which could be found on the sequenced BAC clone AM-BAC-14 (accession number AB525198, Figure 5A). Based on these genes, it is clear that AM-toxin is produced by a NRPS using 2-hydroxyisovalerate, 2-amino-*p*-hydroxyphenylvalerate (Ahv), and two alanines (Figure 5B). The 2-hydroxyisovalerate is generated from 2-ketoisovalerate via the reductase AMT2. Ahv is generated from tyrosine following two cycles of one carbon homologation, akin to leucine biosynthesis from valine (Kohlhaw, 2003). Specially, the transaminase AMT5 converts tyrosine into the corresponding α-ketoacid. AMT7, a 2-isopropylmalate synthase, adds acetyl-CoA to the 2-keto acid, and the product is isomerized by AMT8, an aconitase, to generate the precursor for AMT6, a 3-isopropyl malate dehydrogenase, which effects oxidative decarboxylation generating the α-ketoacid of homotyrosine (Yue et al., 2015). Reiteration of this one-carbon homologation catalyzed by AMT7, AMT8, and AMT6 generates the 2-keto-*p*-hydroxyphenylvalerate, which is converted into the α-amino acid via AMT5 producing Ahv. These enzymatic reactions provide access to the key building blocks required for AM-toxin biosynthesis.

As AM-toxin is a cyclic tetrapeptide, the NRPS responsible for its production, AMT1, must incorporate four amino acids, however examination of its domain architecture shows it lacks an adenylation domain in the final elongation module. Homologous to basidiomycete type VI siderophore synthetases (Brandenburger et al., 2017), AMT1 possesses an A_3_-T_3_-C_4_-T_4_-C_t_ architecture. Based on this similarity, A_3_ is predicted to amino acylate both T_3_ and T_4_ with alanine. Thus A_1_ is selective for 2-hydroxyisovalerate, A_2_ for Ahv, and A_3_ for alanine. The completed tetrapeptide linked to T_4_ is then cyclized by the offloading C_t_ domain (Gao et al., 2012). The cyclic tetrapeptide is subsequently O-methylated by AMT9 and presumably oxidized by the cytochrome p450 to generate the dehdyroalanine residues. This late stage installation of the dehydroalanine residue through oxidation is consistent with the AM-toxin NRPS and supported by the observation that the fusaristatin biosynthetic gene cluster also possess a cytochrome p450 (FGSG_08207), which may convert an alanine residue into a dehydroalanine on the macrocycle (Sørensen et al., 2014). However, biochemical confirmation of this biosynthesis is still required.

The AM-toxin biosynthetic gene cluster has clearly been localized to an AC. The *AMT1* gene was shown by electrophoretic karyotyping and Southern blot probe labeling experiments to localize to ACs of 1.1–1.7 Mb in size (where AC size was dependent on the *A. alternata* apple pathotype strain examined). Furthermore, the *AMT1* gene is absent in non-pathogenic strains (Akamatsu et al., 1999; Johnson et al., 2001). Loss of the associated ACs coincided with the loss of *AMT1* gene expression, resulting in an observed AM-toxin-minus phenotype in a leaf necrosis bioassay (Johnson et al., 2001).

## Linking Secondary Metabolism With ACs in the Next Generation Sequencing Era

The routine accessibility of long-read or single-molecule sequencing platforms has catalyzed an explosion of highly contiguous fungal genomes in recent years. Platforms developed by Oxford Nanopore Technologies and Pacific Biosciences (PacBio) can effectively resolve genomic regions of low complexity and/or highly repetitive content, producing “closed” or “telomere-to-telomere” assemblies. The quality of fungal genome assemblies is often improved by inclusion of massively parallelized, high-fidelity shorter read sequences (such as those derived from Illumina platforms) in assembly pipelines. The resulting closed genomes are highly desirable for the study of rearrangements, regulation of clustered genes, gene duplications, transposable element proliferation and other forms of gene migration between genome compartments such as ACs, accessory regions and core chromosomes (Thomma et al., 2016).

To explore relationships between secondary metabolite biosynthesis and ACs, we compiled 15 recently published fungal genomes containing sequences identified as ACs and scanned them using the fungal version of antiSMASH (Blin et al., 2019) to detect secondary metabolite biosynthetic gene clusters (Table 1). Most of the genomes included in the analysis were produced using long-read sequencing; however, we included three genomes obtained by short-read sequencing platforms where chromosomes were identified as “lineage-specific chromosomes” or “minichromosomes” by optical mapping, electrophoretic karyotyping and/or strain- or species-specific genome comparisons (Ma et al., 2010; O’Connell et al., 2012; Wiemann et al., 2013). When available, gene predictions from the literature were used and in cases where no predictions were available, genes were predicted using antiSMASH default fungal parameters (see Supplementary Table S1 for predicted gene locations). Fungal taxa investigated included representatives from the genera *Alternaria, Colletotrichum, Fusarium, Magnaporthe*, and *Zymoseptoria*.

**TABLE 1:** Secondary metabolite biosynthetic gene clusters (BGCs) predicted from recently published fungal accessory chromosome (AC) sequences.

Taxon/Strain ID	Assembly accession	Sequencing platform	BGC type	Predicted product	AC-associated sequence ID	Core biosynthetic gene ID	Source
***Alternaria***							
*A. alternate* Z7 tangerine pathotype	GCA_001572055.1	PacBio + lllumina	NRPS + PKS	AC-toxin**	Contig 48	AALTg_11749-50	Wang et al., 2016
							Wang et al., 2019
			PKS	PR-PKS	Contig 48	AALTg_11757	
			PKS	PR-PKS**	Contig 58	AALTg_11954	
			PKS	PR-PKS**	Contig 35	AALTg_11074	
*A. brassicae* J3	GCA_004936725.1	Nanopore	PKS	PR-PKS(Ψ)**	ABRSC11	AbrJ3_AS_gene1	Rajarammohan et al., 2019
			PKS	PR-PKS(Ψ)**	ABRSC11	AbrJ3_AS_gene2	
			PKS	PR-PKS**	Scaffold 13	AbrJ3_AS_gene3	
			PKS	PR-PKS(Ψ)**	Scaffold 16	AbrJ3_AS_gene4	
			NRPS	HC-toxin**	Scaffold 16	AbrJ3_AS_gene5	
			NRPS	HC-toxin**	Scaffold 17	AbrJ3_AS_gene6	
*A. alternata* FERA 650 asian pear pathotype	GCA_004156025.2	Nanopore + lllumina	PKS	AK-toxin (AFT16)	Contig 14	AG0111_0g11742	Armitage et al., 2020
			PKS	AF-toxin AFT9-1**	Contig 14	AG0111_0g11753	
			NRPS-like	Unknown	Contig 14	AG0111_0g11815	
			PKS	HR-PKS	Contig 16	AG0111_0g12194	
			PKS	AF-toxin AFT9-1**	Contig 24	AG0111_0g13145	
*A. alternata* FERA 1166 apple pathotype	GCA_004156035.1	Nanopore + lllumina	NRPS-like	Unknown**	Contig 15	AA0116_g12891	Armitage et al., 2020
			NRPS-like	Unknown**	Contig 14	AA0116_g12671	
			PKS	PR-PKS**	Contig 14	AA0116_g12644	
			PKS	PR-PKS(Ψ)**	Contig 15	AA0116_g12873	
			PKS	PR-PKS**	Contig 18	AA0116_g13374	
			NRPS-like	Unknown	Contig 20	AA0116_g13471	
			NRPS	AM-Toxin**	Contig 20	AA0116_g13459	
			NRPS	AM-Toxin**	Contig 21	AA0116_g13506	
***Fusarium***							
*F. fujikuroi* IMI 58289	GCF_900079805.1	Roche 454	CDPS	CDPS	Chr12	FFUJ_14129	Wiemann et al., 2013
*F. poae* 2516	GCA_001675295.1	PacBio + lllumina	PKS	Gibepyrone (PKS8)*	Contig 100	FPOA_12973	Vanheule et al., 2016
			PKS	HR-PKS *(PKS2)*	Contig 165	FPOA_13908	
			Terpene	Koraiol*’**	Contig 109	FPOA_13095	
			Terpene	Koraiol*’**	Contig 110	FPOA_13097	
			Terpene	Koraiol*’**	Contig 126	FPOA_13378	
			Terpene	Koraiol*’**	Contig 131	FPOA_13412	
			Terpene	Koraiol*’**	Contig 134	FPOA_13441	
			Terpene	Koraiol*’**	Contig 26	FPOA_12190	
			Terpene	Koraiol*’**	Contig 51	FPOA_12425	
*F. poae Fp*157	WOUF00000000	Nanopore + lllumina	PKS	HR-PKS (PKS2)*	Contig_2	FPOAC1_013615	Witte et al., 2021
			NRPS	Unknown (NRPS4-Ψ)*	Contig_2	FPOAC1_013344 + 014147	
			NRPS	Apicidin *(NRPS31)*	Contig_3	FPOAC1_013755	
			Terpene	Koraiol*’**	Contig_1	FPOAC1_012967	
			Terpene	Koraiol*’**	Contig_3	FPOAC1_013813	
*F. oxysporum f. sp. conglutinans Fo*5176	GCA_000222805.1	PacBio + lllumina	PKS	HR-PKS *(PKS22)*	Chr3	Fo5176_AS_gene1	Fokkens et al., 2020
*F. oxysporum f. sp. lycopersici Fo*4287	GCA_000149955.2	Sanger + optical mapping	PKS	HR-PKS (PKS22)*	Chr3	FOXG_14850	Ma et al., 2010
			NRPS	Unknown *(NRPS40*)	Chr14	FOXG_17272	
			Terpene	Squalene-hopene cyclase	Chr14	FOXG_17453	
							
*F*. *oxysporum f. sp. radicis-cucumerinum Forc*016	GCA_001702705.2	PacBio	NRPS-like	Unknown	ChrRC	AU210_015923	van Dam et al., 2017
**Other taxa**							
*Colletotrichum higginsianum*	GCF_001672515.1	Sanger + lllumina + optical mapping	PKS	HR-PKS	Chr12	CH63R_14522	O’Connell et al., 2012
IMI 349063							
							

*Synthase/synthetase is paralogous to core chromosome gene (nucleotide ID > 70%, coverage > 95%). **Synthase/synthetase is multiplicated on predicted accessory chromosome (nucleotide ID > 90%, coverage > 95% except where pseudogenized—Ψ) Names in brackets indicate matches to Fusarium synthase/synthetase clades as described in Hansen et al. (2015) and Brown and Proctor (2016). HR-PKS, highly reducing polyketide synthase; PR-PKS, partially reducing PKS; NRPS, non-ribosomal peptide synthetase; CDPS, cyclodipeptide synthase.

Most of the biosynthetic gene clusters associated with ACs in this subset of fungi are encoded by isolates of *Fusarium* and *Alternaria*, an unsurprising fact as these are genera associated with diverse secondary metabolism products and are likely over-represented in available databases of high-quality genomes. Published genomes derived from isolates of *Zymoseptoria tritici, Magnaporthe oryzae, Colletotrichum graminicola, Fusarium oxysporum* f. sp. *lini* and *Fusarium solani* (anamorph of *Nectria haematococca)* all lacked AC-associated secondary metabolite biosynthetic gene clusters, whereas *Alternaria* and *Fusarium* genomes contained numerous secondary metabolite biosynthetic gene multiplications in AC-associated sequences. The gene multiplication exhibited on ACs suggests that ACs are fertile grounds for secondary metabolite biosynthetic gene amplification and neofunctionalization (discussed below).

Our analysis underlines the utility of long-read sequencing in associating biosynthetic gene clusters to AC sequences in fungi. This marks an important shift in the study of AC-associated secondary metabolism, which can now be inferred and characterized without prior study of phenotypic or biological association. Three fungal secondary metabolites were recently attributed to biosynthetic gene clusters on AC-associated sequences thanks to long-read sequencing efforts. HC-toxin and apicidin are two structurally related cyclic tetrapeptides encoded by AC-associated biosynthetic gene clusters in *A. brassicae* strain J3 and *F. poae* strain *Fp*157, respectively (Rajarammohan et al., 2019; Witte et al., 2021), and koraiol is the product of a terpene synthase/cyclase detected in numerous *Fusarium* AC sequences (Table 1). The functional role of these secondary metabolites as virulence factors in the context of *Alternaria* and *Fusarium* host pathogenicity is currently unknown. Both HC-toxin and apicidin are known histone deacetylase inhibitors associated with potent bioactivity and their associated presence with ACs is likely not trivial to the evolution of fungal plant pathogenicity.

### Apicidins

The apicidin family of non-ribosomal peptides are produced by various *Fusarium* species, including *F. semitectum*, *F. sambucinum*, and *F. fujikuroi* (Darkin-Rattray et al., 1996; Singh et al., 1996, 2001, 2002; Park et al., 1999; Jin et al., 2008, 2010; Von Bargen et al., 2013; Niehaus et al., 2014). The apicidins are potent, reversible histone deactylase inhibitors (Darkin-Rattray et al., 1996) that show antiprotozoal activity against *Plasmodium berghei* and *P. falciparum* (Darkin-Rattray et al., 1996) and induce apoptosis in human leukemic HL-60 cells (Kwon et al., 2002). Histone deacetylase inhibition can lead to hyper-acetylation of histones, impacting an organism’s ability to regulate genetic transcription. From *in planta* experiments, apicidins were observed to negatively impact the development of roots in seedlings, resulting in chlorosis and necrosis on leaves, distorted leaf shape in developing leaves, and stunted plant growth (Jin et al., 2008). Exposure of duckweed to apicidins caused an inhibition of cell division, cellular leakage, and an impairment of chlorophyl synthesis due to chloroplast deterioration (Abbas et al., 2001). Apicidins are structurally similar to HC-toxin, another cyclic tetrapeptide histone deacetylase-inhibitor with a well-documented role as a virulence factor during infection by the fungal plant pathogen *Cochliobolus carbonum*.

**FIGURE 6:**
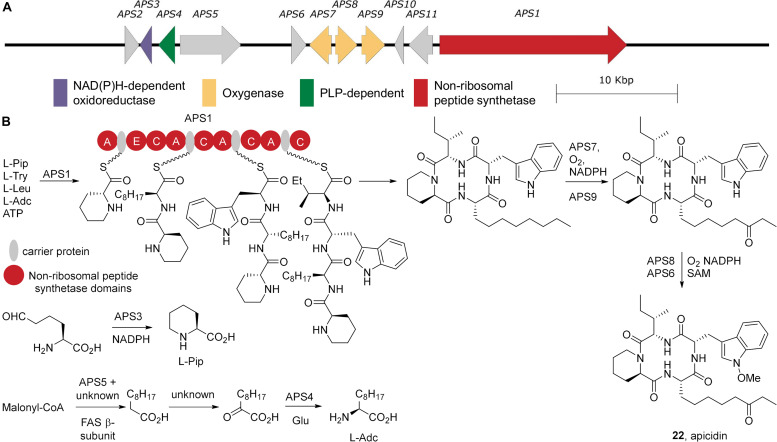
Apicidin biosynthesis. **(A)** The apicidin biosynthetic gene cluster from *Fusarium incarnatum.* **(B)** The biosynthesis of apicidin and its non-proteinogeneic amino acid building blocks. A, adenylation; C, condensation; E, epimerization.

The parent compound apicidin B is a cyclic tetrapeptide comprised of a L-pipecolic acid, an L-isoleucine, a N-methoxy-L-tryptophan, and a L-8-keto-2-aminodecanoic acid residue (Figure 6). The biosynthetic gene clusters responsible for apicidin production in *F. semitectum* and apicidin F production in *F. fujikuroi* have been identified (Jin et al., 2010; Niehaus et al., 2014) and both clusters show high nucleotide identity over the coding regions of the assigned biosynthetic genes (>60%). Bioinformatic based comparisons show that the gene clusters are also present in the genomes of *F. incarnatum* and *F. scirpi* (Villani et al., 2019). The building blocks for the NRPS-mediated biosynthesis of apicidin are the proteinogenic amino acids L-isoleucine and L-tryptophan. The non-proteinogenic amino acid L-pipecolic acid (L-Pip) and L-2-aminodecanoic acid (L-Adc) must be generated specifically for apicidin biosynthesis. *APS3* encodes a reductase responsible for converting 2-aminoapidate semialdehyde into L-Pip. Consistent with this, deletion of *APS3* leads to the production of apicidin B, an analog where the L-Pip is replaced with L-proline (Jin et al., 2010). The genes encoding L-Adc formation are not all present in the apicidin biosynthetic gene cluster. A fatty acid synthase α-subunit, *APS5* is present and is required for apicidin production, however the corresponding β-subunit is absent in the biosynthetic gene cluster and is likely located elsewhere in the genome. These synthases likely generate decanoate, which is further elaborated via α-oxidation by an as yet uncharacterized set of enzymes into 2-ketodecanoate. The aminotransferase *APS4* then coverts this into L-Adc. *APS1*, the NRPS then epimerizes and condenses the L-Pip to L-Adc, L-tryptophan, and L-isoleucine, followed by cyclization via its C-terminal domain (Gao et al., 2012). The tryptophan and Adc side chains are then oxidized, though the order of oxidation is unclear. C8 oxidation of Adc via the monooxygenase *APS9*, generates an *S*-configured alcohol, which is followed by the oxidation to the ketone by the cytochrome p450 *APS7* (Jin et al., 2010). N-oxidation of the tryptophan by *APS8* (Niehaus et al., 2014) followed by O-methylation via *APS6* (Jin et al., 2010) completes the biosynthesis.

The biosynthetic gene cluster associated with apicidin F production is localized to the end of chromosome I of *F. fujikuroi* as determined through analysis of a high quality, 12 scaffold genome that was assembled from pyrosequencing data (N50 = 4.2 Mb) (Wiemann et al., 2013). The position of the gene cluster at the chromosome end suggests it may have been acquired by horizontal gene transfer as end regions are more prone to recombination than telomere-distal regions. Consistent with horizontal acquisition, other *Fusarium* spp. evolutionarily related to *F. fujikuroi* do not produce apicidins (Niehaus et al., 2014). Examination of 13 *Fusarium incarnatum-equiseti* species complex genomes showed the apicidin biosynthetic gene cluster was present in only one member of the Incarnatum clade and two members of the Equiseti clade (Villani et al., 2019). A recent, high-quality genome strongly suggests the apicidin gene cluster resides on an AC in at least one isolate of *F. poae* (Witte et al., 2021).

## HC-Toxin (Predicted in *A. brassicae*)

Like apicidin, HC-toxin is a cyclic tetrapeptide and contains an oxidized L-Adc residue. It is also a histone deacetylase inhibitor, inhibiting the RPD3 class histone deacetylases, which affects root growth of susceptible maize cultivars (reviewed in Walton, 2006). Highly virulent *C. carbonum* strains contained a unique genetic locus (collectively designated as *TOX2*) that spans a 500kb region and is characterized by highly repetitive sequences in which multiple, functional copies of *TOX2* reside (Walton, 2006). The *TOX2* locus contains the core HC-toxin biosynthetic genes, including the gene encoding the non-ribosomal peptide synthetase HCT1 (Scott-Craig et al., 1992) and shows sequence homology to a number of the apicidin biosynthetic genes (Stergiopoulos et al., 2013).

**FIGURE 7:**
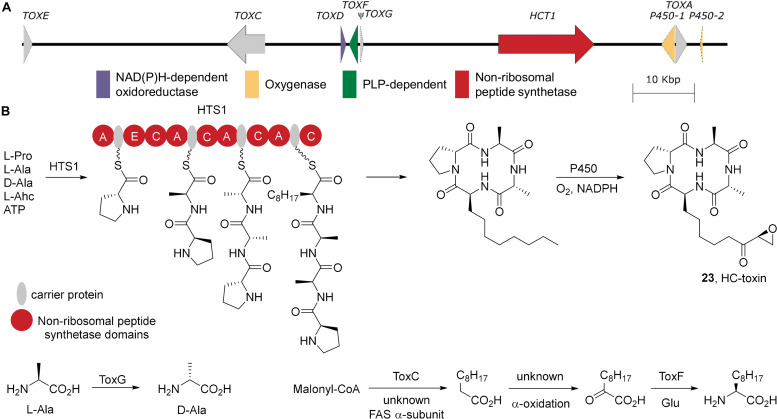
HC-toxin biosynthesis. **(A)** The biosynthetic gene cluster for HC-toxin from *A. brassicae* strain J3 scaffold 18 (SMOM01000016.1). **(B)** The biosynthesis of HC-toxin and its non-proteinogenic building blocks. A, adenylation; C, condensation; E, epimerization.

Characterization of the HC-toxin biosynthetic gene cluster from *TOX2* has proven challenging. The biosynthetic genes are sparse in this region of the genome and most occur as multiple functional copies. Furthermore the biosynthetic genes are embedded in highly repetitive sequences (Walton, 2006). While *TOX2* has been well mapped in *C. carbonum* (Ahn and Walton, 1996; Ahn et al., 2002), no complete physical map is available of the HC-toxin gene cluster. However a recent complete genome sequence of *A. brassicae* revealed a putative HC-toxin biosynthetic gene cluster on an AC (Rajarammohan et al., 2019; Figure 7A). Unfortunately the genome assemblies of both *A. jesenskae* and *C. carbonum* are as yet too fragmented to determine whether the *TOX2*/*TOX2*-like clusters are localized to ACs or core chromosomes in those species. The detection of a *TOX2*-like cluster on an AC in *A. brassicae* has important implications for the evolution of virulence in this genus, particularly since HC-toxin is well established to be an important virulence factor in *C. carbonum* strains infecting maize genotypes (for review see Walton, 2006).

HC-toxin is produced by the NRPS HCT1 which utilizes L-proline, L-alanine, D-alanine, and L-Adc as building blocks (Figure 7B). The first A domain of the NRPS activates L-proline and loads it onto the carrier protein where it is epimerized generating D-proline. The second A domain loads L-alanine onto the following carrier protein and the C domain condenses with D-proline. The third A domain is presumably D-alanine selective. D-Alanine is generated via TOXG, an alanine racemase, from L-alanine (Cheng and Walton, 2000). Intriguingly, in *A. brassicae TOXG* is pseudogenized. However it is documented in *C. carbonum* that *TOXG* mutants can produce a variant of HC-toxin where D-alanine is replaced by glycine (Cheng and Walton, 2000). Thus, it seems likely that this *A. brassicae* strain generates the glycine variant of HC-toxin. The growing peptidyl intermediate D-proline-L-alanine is then coupled to a carrier protein bound D-alanine. Lastly, the final A domain likely activates L-Adc and couples this to complete the linear enzyme bound tetrapeptide. Similar to the apicidin biosynthetic gene cluster, the HC-toxin biosynthetic gene cluster contains only a subset of the genes responsible for the formation of L-Adc. *TOXC* encodes a fatty acid synthase β-subunit which is required for HC-toxin production (Ahn and Walton, 1997) presumably by generation of the medium chain fatty acid for L-Adc formation. However, no corresponding α-subunit has been identified. Oxidation to the 2-ketodecanoate is also required though, as of yet, no genes responsible for the oxidation have been identified. *TOXF* encodes a putative branched-chain amino acid transaminase (Cheng et al., 1999), which can convert the 2-ketodecanoate into L-Adc. The terminal C domain then completes the cyclization of the linear enzyme-bound tetrapetidyl group. Cytochrome p450-mediated oxidation of the L-Adc side chain then generates the completed, highly bioactive natural product. In *A. brassicae*, there appear to be multiple cytochrome p450s closely associated with *TOXA*, which encodes the major facilitator superfamily membrane transporter responsible for secretion of HC-toxin and self-protection (Pitkin et al., 1996).

## Koraiol

**FIGURE 8:**
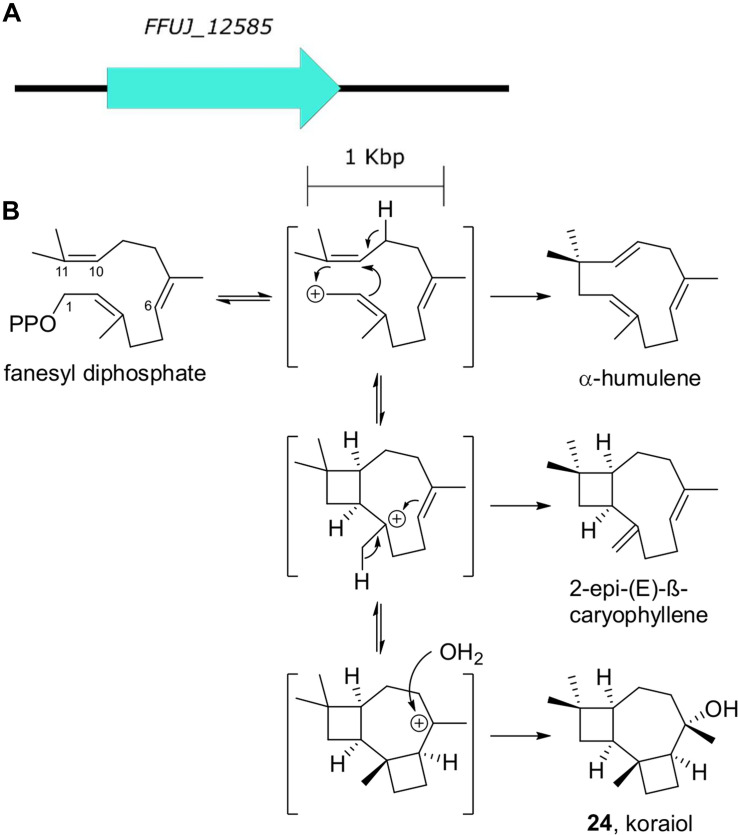
Koraiol biosynthesis. **(A)** The terpene synthase gene responsible for koraial biosynthesis. **(B)** Farnesyl diphosphate cyclization generates koraiol as well as additional 1,11-cyclized sequiterpene natural products.

Koraiol is a tricyclic sesquiterpene alcohol structurally related to caryophyllene. It has been shown to be produced *in vitro* from the class I terpene synthase *STC4* from *Fusarium fujikuroi* that effects a 1,11 cyclization of the primary metabolite precursor farnesyl diphosphate (Brock et al., 2013; Figure 8). While a variety of fungal sesquiterpene synthases are known, phylogenetic analysis suggest that the synthases that generate the 1,11-cyclized products form a clade that is separate from the synthases that generate 1,6-cyclized sequiterenoids like trichodienes and the 1,10-cyclized sequiterpenes like aristolochene (Wawrzyn et al., 2012; Quin et al., 2014).

*STC4* homologs are commonly detected in *Fusarium* genomes (Niehaus et al., 2016; Hoogendoorn et al., 2018; Villani et al., 2019). A “multi-omics” study comparing secondary metabolite biosynthetic gene cluster expression and chemical phenotypes of *Fusarium* species detected *STC4* expression in *F. mangiferae, F. fujikuroi, F. verticillioides*, and *F. proliferatum* isolates cultured both *in vitro* and in maize root infection experiments, but only detected koraiol from *F. fujikuroi* metabolomes (Niehaus et al., 2016). While the effect of koraiol on plant-fungal interactions is unclear, Niehaus et al. speculate that high levels of terpene cyclase expression in all growth conditions tested supports a basic growth- or interaction-associated role of koraiol which is unlikely to be host-specific.

## Acs Are Linked With Secondary Metabolite Biosynthetic Gene Duplications and Disruption

Biosynthetic gene multiplicity associated with ACs drives the evolution of pathogenicity. From our comparison of hybrid long and short read genomes (Table 1), a considerable number of multiplicated genes were found to be associated with ACs, including koraiol synthase, HC-toxin synthetase, AF- and AM-toxin synthetases, and other predicted genes that have yet to be associated with secondary metabolite products. In *Alternaria* pathotypes, the presence of multiple copies of biosynthetic genes on ACs results in an amplification of host specific toxin production (Izumi et al., 2012a, b). Harimoto et al. (2007, 2008) found that the wild-type AM-toxin producing pathotype of *A. alternata* (strain IFO8984) likely has 3 or 4 copies of AMT2, AMT3 and AMT4, and observed that mutants containing single and double copies of AMT2 and AMT3 produced less AM-toxin than the wild-type strain. The single and double-mutants of AMT gene knockouts were confirmed as less virulent in plant infection trials compared to wild-type. These observations underline the importance of gene duplication as a potential mechanism to increase host specific toxin production in aggressive pathotypes. Other duplicated *Alternaria* secondary metabolite biosynthetic genes include those involved in AK-toxin production in *A. alternata* pear and apple pathotypes (Armitage et al., 2020). Interestingly, biosynthetic gene cluster duplications observed on ACs from *A. alternata* strains differ from duplications observed in *Fusarium* spp. In *Fusarium*, many AC-associated biosynthetic genes are slightly divergent paralogs of core chromosome genes, including synthase/synthetases *PKS2, NRPS4, PKS8* (predicted to encode gibepyrone synthase), and *PKS22* (for *Fusarium* biosynthetic enzyme clade nomenclature see Hansen et al., 2015 and Brown and Proctor, 2016). These AC/core chromosome paralogous pairs generally share 70–90% nucleotide identity, suggesting they are either rapidly diverging or are the products of relatively older duplication events.

Not all *Fusarium* AC-associated biosynthetic gene clusters are mere duplicates of core chromosome-associated biosynthetic gene clusters. The sesquiterpene cyclase gene *STC4*, associated with koraiol production (Figure 7), is multiplicated six times in *F. poae* strain 2516 and twice on ACs in *F. poae* strain *Fp*157 (Vanheule et al., 2016; Witte et al., 2021). Four of the *F. poae* 2516 *STC4* copies were heterologously expressed in yeast and three were shown to encode functional koraiol synthases, supporting the functionality of the duplicates (Hoogendoorn et al., 2018). Hoogendoorn et al. point to *STC4* copies as supporting the classic view of duplication as a means of relaxing evolutionary pressure on unigenes to enable the evolution of novel function (Ohno, 1970; Taylor and Raes, 2004). Another possibility is that more *STC4* copies could simply mean more koraiol production, as is the case with duplicated *Alternaria* biosynthetic genes. Furthermore, the detection of an *STC4* paralog in a subtelomeric region of *Fp*157 core chromosome 1 with a 98% nucleotide identity match to the two AC-associated koraiol synthase gene copies found from the same strain (Witte et al., 2021) indicates that gene transfer is likely happening between core and accessory chromosomes. Inter-chromosomal gene transfer could be an important mechanism by which successful combinations of biosynthetic genes and regulatory elements rapidly evolve on ACs and then move to core genome regions associated with higher levels of meiotic stability.

**FIGURE 9:**
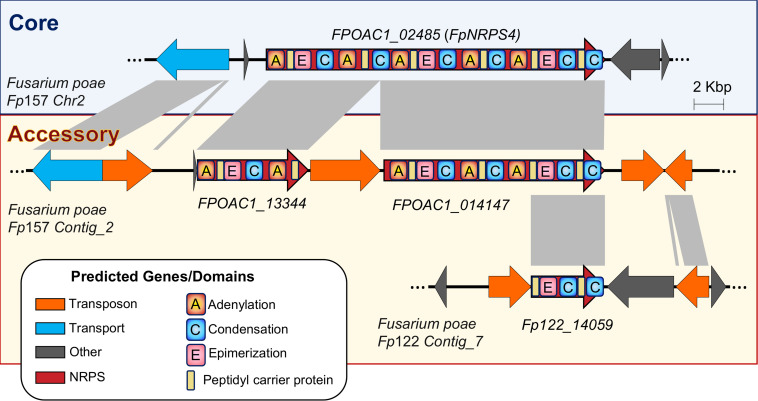
Accessory chromosomes in *Fusarium poae* illustrate the potential for biosynthetic gene duplication and TE-mediated truncation from which new small molecules could be produced.

In addition to duplication, gene disruption via active transposon insertions and rearrangements appears common to both *Alternaria* and *Fusarium* biosynthetic gene clusters, where synthase or synthetase copies have missing domains crucial to the production of small molecules (Table 1). In PKSs and terpene synthase/cyclases this disruption likely leads to pseudogenization, however given the modular nature of NRPSs, the potential for truncated—but still functional—NRPS genes producing new molecules is worth investigation. Concrete examples of this are lacking at present, however *F. poae NRPS4*, the product of which is as yet uncharacterized, illustrates how TE disruption-associated truncation could happen (Figure 9). As more long-read genomes become available, we detect additional variations on truncated NRPSs in *F. poae*. Truncation followed by rearrangement could, however infrequent, lead to new combinations of domains and be an important process in the evolution of fungal secondary metabolite biosynthesis.

## Role of Repeat-Induced Point Mutation (RIP) Mechanisms

**FIGURE 10:**
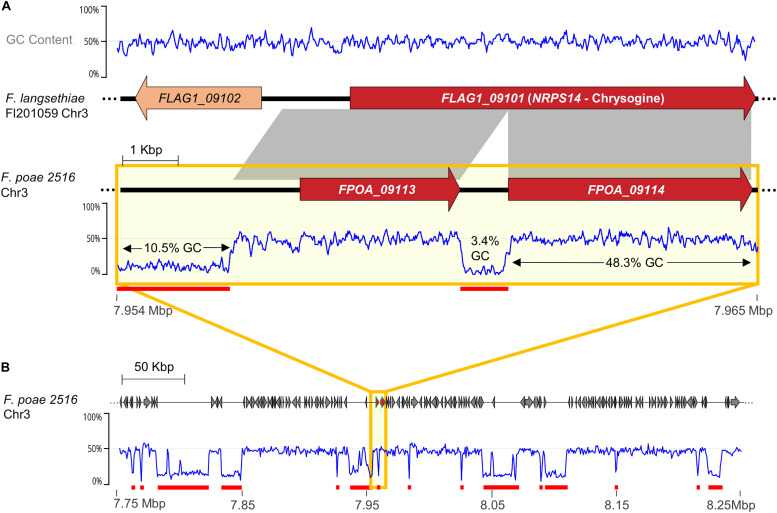
**(A)** Comparison of chrysogine synthetase *NRPS14* homologs in *F. langsethiae* Fl 201059 and *F. poae* 2516, illustrating genomic regions predicted to be heavily influenced by repeat-induced point mutation (RIP). Gray blocks indicate syntenic regions (>90% nt identity). *F. langsethiae* is a known chrysogine producer, whereas *F. poae* is not, likely because *NRPS14* has been pseudogenized via transposable element insertion. The offending transposable element was then likely subject to RIP, leaving an approximately 800 bp long region of very low GC content (3.4%). **(B)** Overview of surrounding genetic region on Chr3 showing numerous regions of very low GC content. Red underscores indicate sequence blocks with very low average GC content (<20%).

Genetic duplication is widely held to be a fundamental prerequisite for the evolution of novel function by relaxing selective pressure on individual genes to enable mutation accumulation (Ohno, 1970; Taylor and Raes, 2004). However, duplications involving rapidly multiplying transposable elements can also be disruptive and appear parasitic in nature. “Repeat Induced Point-mutation” (RIP) is a genome defense mechanism that acts to control such parasitic duplications. Activated during the pre-meiotic stage of the sexual cycle, RIP inactivates duplicated genes over a certain length, via induced nucleotide mutation and epigenetic silencing (Selker et al., 1987; Selker and Garrett, 1988; Galagan and Selker, 2004). Since its discovery in *Neurospora crassa*, RIP has been detected in many filamentous ascomycetes including many important plant pathogens (Ikeda et al., 2002; Idnurm and Howlett, 2003; Cuomo et al., 2007; Clutterbuck, 2011), and is considered a powerful tool to maintain genetic integrity. RIP activity is typically inferred by scanning a genome for the presence of regions over a certain base pair length that are GC-poor compared to background codon frequencies, since RIP converts G:C to T:A dinucleotides in duplicated regions. The scars of disruptive, RIP-subdued transposable elements can be observed as low GC-content sequences inserted into coding regions, as appears to be the case for the pseudogenized chrysogine NRPS present in *F. poae* genomes (Figure 10).

While RIP is clearly advantageous for the defense of genomes, it also likely suppresses gene duplications considered essential for adaptive evolution, including biosynthetic gene cluster duplication (Galagan and Selker, 2004). This presents a paradox: how can plant pathogenic fungi promote adaptation to plant defenses with reduced gene duplication? One solution to the problem could be to stop the sexual cycle and thereby stop RIP. This concept is consistent with the lack of observed sexual states in many plant pathogenic fungi that harbor ACs, including *F. poae*, *F. oxysporum* and *A. alternata*. Alternatively, genomes could be compartmentalized such that RIP is restricted to a subset of chromosomes or chromosome regions. This solution is consistent with the presence of intact, clustered tandem rDNA repeats in genomes of RIP-active species (*L. maculans* excepted), and is also consistent to *F. poae* genomes, which have less evidence for RIP in ACs than in core chromosomes (Vanheule et al., 2016; Witte et al., 2021). Little has been reported on RIP in *Alternaria*, although it has been computationally predicted to occur in at least one species (Clutterbuck, 2011). More research is needed to determine whether the presence of numerous duplicated biosynthetic genes on *Alternaria* ACs is related to RIP inactivation or genome compartmentalization. Importantly, the mere presence of ACs does not imply RIP activation or inactivation: *Zymoseptoria tritici*, for example, shows clear evidence for RIP in its ACs (Testa et al., 2015), and *Leptosphaeria maculans* has an AC predicted to be over 90% affected by RIP (Rouxel et al., 2011). During our long read genome analysis (Table 1), we did not detect secondary metabolite biosynthetic gene cluster duplications in genomes of either species. More research is needed to explore the idea of genetic compartmentalization and ACs in RIP-active species, and could be helpful in defining the functional differences between ACs and accessory regions of core chromosomes.

## Linking ACs and Accessory Regions of Core Chromosomes

Our understanding of how ACs originate and affect core chromosome evolution is growing. The potential for gene cluster translocation between core and ACs (often localized in “accessory regions” usually proximal to telomeres) is relevant. Comparative genomic analysis of *Zymoseptoria tritici* genomes in isolates undergoing sexual reproduction supports an origin for some ACs via chromosome duplication, fusion and degeneration, which involve “breakage-fusion-bridge” cycles between chromosomes followed by mutational decay (Croll et al., 2013). In asexual species, however, the origins of ACs and their relationship to core chromosomes is less resolved. Both types of chromosomes can share genetic content, including transposon “invasions” from accessory chromosomes into core chromosomes (Vanheule et al., 2016), and other gene duplications as described above. Gene cluster translocation from an AC to a core chromosome is an interesting possibility that requires more study. In some *F. poae* isolates, an accessory region was found to contain lineage-specific, 100 Kb + sequence blocks bordered by unusual transposons (Vanheule et al., 2016). These regions were proposed to represent integration of AC sequences into core chromosomes (Vanheule et al., 2016). Interestingly, genes with “DUF3435” domains, implicated in large sequence translocations in *Podospora* genomes, have been detected in *F. poae* 2516 and are a promising avenue for further research of large translocations between chromosomes (Vogan et al., 2020). Understanding how genes move between chromosomes will illuminate the potential structural ramifications of the presence of ACs within a genome.

## Implications of Horizontal Chromosome Transfer of ACs

Horizontal transference of an AC between fungal strains can turn an endophyte or saprophyte into a pathogen (Ma et al., 2010; Vlaardingerbroek et al., 2016a; van Dam et al., 2017), suggesting important implications for the evolution of asexual plant pathogens. Compared to core chromosomes, genomic content of ACs are relatively dynamic (Vlaardingerbroek et al., 2016b; Habig et al., 2017; Möller et al., 2018; Plaumann et al., 2018). ACs can change in size during horizontal chromosome transfer, as higher concentrations of repetitive elements in ACs lead to intra- or interchromosome homologous recombination, resulting in deletions, translocations (Hedges and Deininger, 2007) and multiplication of gene loci (Li et al., 2020). Although exact mechanisms for horizontal chromosome transfer have yet to be fully elucidated, current hypotheses involve AC transfer events through heterokaryosis during hyphal anastomosis (Bertazzoni et al., 2018). *In planta*, we hypothesize that anastomosis may arise when intracellular hyphae of two strains interact in close proximity in the apoplast of host cells (especially in pathogens that exhibit an endophytic or biotrophic phase). The ability to maintain ACs with virulence factors in a subset of a fungal population could permit the maintenance of different trophic phases within the species, providing an adaptive advantage in periods of time when plant populations develop countermeasures to infection.

## Core and Accessory Chromosome Regulation of Secondary Metabolite Gene Cluster Expression

In fungi, secondary metabolism is regulated by various, often overlapping mechanisms that include pathway-specific and global regulators, signal transduction pathways, and epigenetic control (Macheleidt et al., 2016). Global regulators translate environmental cues to induce the transcription of multiple secondary metabolite pathways while pathway-specific transcription factors typically only control transcription of their respective cluster biosynthetic genes. Genes for cluster-specific regulators can be located either outside or inside of the cluster that they regulate, a fact that has implications on gene cluster expression or “cross talk” in organisms with ACs. Genes on acquired ACs may not have promoter sequences targeted by global regulators native to the host’s core chromosomes. Therefore, for AC-associated gene cluster expression in the recipient strain to be assured, appropriate transcription factors need to either be encoded on the AC or be sufficiently conserved in their DNA binding domains if encoded on the core genome (van der Does et al., 2016). Additionally, if multiple copies of conserved global regulators reside on ACs, transcriptional control over biosynthetic gene clusters on core chromosomes might also become regulated from the AC. For example, expression of the transcription factor *EBR1* is involved with the onset of pathogenicity (as a master regulator of primary and secondary metabolism expression) and typically occurs as a single copy on a core chromosome; however, multiple duplications of *EBR1* occur on ACs of *F. poae* and most *F. oxysporum f.*sp. pathogenic strains (Jonkers et al., 2014; Vanheule et al., 2016). The occurrence of multiple transcription factor duplications on ACs may offer a selective advantage through increased expression of particular virulence factors during infection. Similar to core chromosomes, expression of AC-associated secondary metabolism is also transcriptionally regulated via epigenetic control over chromatin structure. From studies with *F. oxysporum*, observations suggest that chromatin-mediated regulation of gene expression occurs on ACs (van der Does et al., 2016).

## A Multi-Omics Approach for the Discovery of AC-Associated Secondary Metabolites

As described in this review, ACs in fungal plant pathogens harbor a diverse secondary metabolite biosynthetic potential. ACs can be vehicles for transferring biosynthetic gene clusters between isolates, they can influence gene regulatory systems, and they hold the potential to expand secondary metabolite structural diversity. This can lead to important differences in the metabolomes of isolates within the same species, confounding efforts to classify populations within a species based on “core”-associated genotypes—as evidenced by classification within the species *A. alternata*.

Combining genomics and untargeted metabolomics is a powerful way to screen populations of fungi for secondary metabolite production associated with ACs. Long-read genome sequencing combined with high-fidelity short-read sequencing is a powerful “top down” genomics approach for characterizing and contextualizing fungal genomes at the chromosomal level. In this context, “top down” refers to the ability to detect nearly all secondary metabolite biosynthetic gene clusters in a genome, even if they are silent under laboratory conditions, and derive AC/biosynthetic gene cluster associations. Untargeted metabolomic analysis, however, takes a “bottom up” approach to discovering AC- or accessory region-associated secondary metabolites. Here, chemical extracts of fungal strains are analyzed using high-resolution mass spectrometry to characterize and compare patterns in secondary metabolite production observed within a population to search of unique patterns attributable to sub-sets within the population. Leveraging results obtained from both metabolomics and genomics experiments within a species population allows for the correlation of lineage-specific secondary metabolite products to unique biosynthetic gene clusters residing on ACs.

**FIGURE 11:**
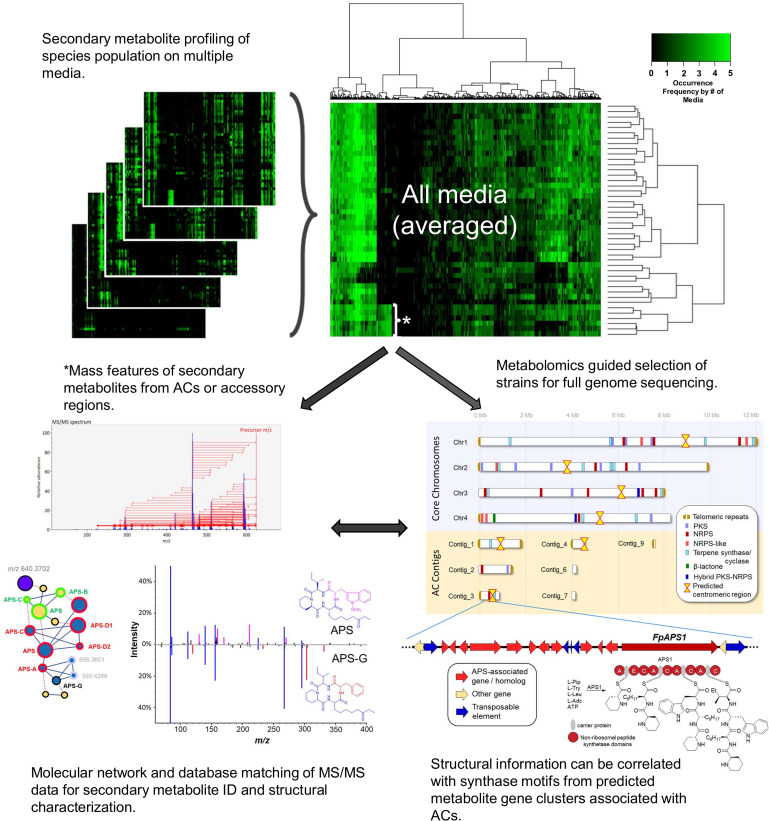
Using metabolomics to identify lineage-specific secondary metabolites produced by active secondary metabolite biosynthetic gene clusters on accessory chromosomes. Extracts from single spore isolates cultured in multiple media conditions are analyzed by high resolution mass spectrometry to produce metabolomes. Metabolomes are averaged across all media to create *in vitro* consensus chemical phenotypes for each strain. Lineage-specific signals (indicated by the asterisk) can be correlated to secondary metabolite biosynthetic gene clusters detected from isolate genomic analysis. Mass spectra can be further analyzed to compare chemical “fingerprints” (MS2 scans) to online spectral databases or *in silico* predictions of molecular fragmentation spectra for compound identification.

This “multi-omics” strategy is currently being applied in studies of agriculturally relevant crop pathogens, making use of strain population libraries generated from annual disease surveys on various crops and geographical locations. In such a strategy, results from metabolomics profiling work are used to make selections of the population for full genome sequencing (long and short-read). For pathogens that are known to have ACs, metabolomes derived from extracts of single spore isolates cultured under axenic conditions on multiple media can be combined to generate “consensus” chemical phenotypes (representing the breadth in secondary metabolism expressed by a given strain; Figure 11). Constructing and comparing “consensus” chemical phenotypes from a species population enables the detection of phenotypic anomalies resulting from transcriptionally active biosynthetic gene clusters found on ACs or subtelomeric accessory regions from core chromosomes. Within the “consensus” phenotype, secondary metabolite associations between core and ACs (or accessory regions) are readily apparent. MS^2^ fragmentation patterns of mass features of interest can be compared to online spectral databases for metabolite identification and molecular networking analysis of MS^2^ spectra applied to detect deviations in the structures of known secondary metabolite products, indicating if new metabolite analogs are present. Structural information of observed secondary metabolite products can then be correlated with synthase/synthetase motifs from genome predicted secondary metabolite biosynthetic gene clusters and associations inferred. Often these inferences are substantiated by follow-up transcriptomic and synthase/synthetase gene knock-out experiments. Further comparison with associated biotic or abiotic metadata (e.g., host specificity, disease phenotypes or geographic location) can promote possible inference regarding the role that AC-acquired secondary metabolite expression may have on the evolution of strain pathogenicity or virulence within the species population.

## Concluding Remarks

The study of the accessory genome is the study of the genomic space in which fungi develop novel invasion strategies and adapt to evolving plant defenses. In some *Alternaria* pathotypes/*f.*sp., ACs act as a chromosomal “engine” for the horizontal transfer, duplication, and rearrangement of secondary metabolite biosynthetic gene clusters. AC-associated gene cluster expression expands both the diversity and quantity of mycotoxins and other virulence factors produced, enabling isolates to invade and adapt to specific plant hosts. Untargeted metabolomics is a promising tool for exploring the evolving chemical space associated with these adaptations. Metabolomic profiling of *Alternaria* and *Fusarium* spp. populations will likely lead to the discovery of more biosynthetically active ACs with potential roles in pathogenicity. Such biosynthetic gene clusters on ACs can also provide insights into the evolution of fungal natural products, as the dynamic nature of ACs can promote diversification of natural product families. Although their effects on pathogenicity are currently unexplored, further study into secondary metabolite biosynthetic gene clusters on *Fusarium* ACs and their resulting products should provide insight into the evolutionary trajectories of closely-related fungal species with divergent lifestyles and genomic architectures, such as *F. poae, F. oxysporum* and *F. graminearum*. The differences between *Alternaria* and *Fusarium* genomes reviewed here suggest there are a multiplicity of relationships between ACs and core chromosomes in fungi, and this diversity will likely expand as more fungal taxa are discovered and sequenced.

## Author Contributions

TW, NV, CB, and DO wrote the manuscript and approved its final version.

## Conflict of Interest

The authors declare that the research was conducted in the absence of any commercial or financial relationships that could be construed as a potential conflict of interest.

## Abbreviations

ACs: Accessory chromosomes
NRPS: Non-ribosomal peptide synthetase
PKS: Polyketide synthase
RIP: Repeat-induced point mutation.
